# Breathability and Moisture Permeability of Cellulose Nanocrystals Hollow Microsphere Coatings for PET Fabrics

**DOI:** 10.3390/polym14245345

**Published:** 2022-12-07

**Authors:** Fan Zhang, Bingyao Song, Yilin Li, Yingying Zhou, Yanbing Wang, Qunna Xu, Jianzhong Ma

**Affiliations:** 1 School of Textile Science and Engineering, Xi’an Polytechnic University, Xi’an 710048, China; 2 Shaanxi Collaborative Innovation Centre of Industrial Auxiliary Chemistry & Technology, Shaanxi University of Science & Technology, Xi’an 710021, China; 3 Key Laboratory of Functional Textile Material and Product, Xi’an Polytechnic University, Ministry of Education, Xi’an 710048, China; 4 College of Bioresources Chemical and Materials Engineering, Shaanxi University of Science and Technology, Xi’an 710021, China

**Keywords:** CNCs, hollow microspheres, breathability, moisture-wicking, polyester fabric

## Abstract

In this study, cellulose nanocrystals hollow microspheres (HMs) were fabricated through Pickering emulsion polymerization, in which hydrophobically modified cellulose nanocrystals (CNCs) acted as Pickering stabilizers. The hollow interior core was prepared by solvent evaporation. This manuscript describes the synthesis of HMs in detail. The hollow structure and nanoscale size of HMs were verified using TEM. The resultant HMs could easily coat self-forming films on the surface of PET fabrics. Additionally, these coatings exhibited superior breathability and moisture permeability properties with a high one-way transport index of 936.33% and a desirable overall moisture management capability of 0.72. Cellulose nanocrystal hollow microsphere coatings could be used as a moisture-wicking functionality agent for finishing fabrics, oil–water separation, and fog harvesting.

## 1. Introduction

Poly(ethylene terephthalate) (PET) fabrics have the advantages of low price, good elasticity, corrosion resistance, stiffness, easy washing and quick drying, etc. [1]. There is a large number of hydrophobic ester groups on the PET molecular chains with very low moisture absorption [2]. The standard moisture regain of PET is around 0.4% [3,4], making it uncomfortable to wear when used as apparel. The breathability and moisture permeability of the fabric are closely related to the objective comfort [5]. Additionally, it is challenging to impart functional characteristics to PET fabrics via chemical modification since there are no polar groups on PET molecular chains other than two terminal alcoholic hydroxyl groups. Therefore, it always needs the help of adhesives to fix the functional auxiliaries on the surface of PET fabrics endowing the surface with different properties, such as antibacterial, anti-ultraviolet, anti-fouling, etc. However, the dense adhesives further hinder the gas penetration, significantly reducing the breathability and moisture permeability of PET fabrics [6]. So, objective comfort properties of polyester fabric can be enhanced by increasing the hydrophilicity and reducing the gas barrier of the fabric [7,8].

In recent years, a substantial improvement in breathability and moisture permeability has been achieved by properly designing both the structure and material for coatings [9]. For example, inspired by living systems, tree-like structures and typical hierarchical structures were designed to effectively transport moisture only from one side to another side [9,10]. Compared with solid polymer materials, hollow materials with a low gas barrier have recently been reported to have excellent gas management performance [11,12]. The structure design has a critical effect on the breathability, while the moisture permeability is more related to the hydrophilic groups on the surface of the coatings. The water molecules (in water vapor) are captured by hydrophilic groups through hydrogen bonds and then pushed from the side in contact with the skin to the surrounding environment. Taking the adhesion into consideration, the development of hollow structured hydrophilic film-forming materials is extremely useful.

For fabrication of hydrophilic film-forming materials with a hollow structure, an approach is to add hollow nanoparticles to the film-forming emulsion. For instance, Bao et al. [13,14] explored films of mesoporous hollow nano-silica polyacrylate emulsion and found that the latex films had the best waterproof and moisture permeability when the cavity diameter of the mesoporous hollow SiO_2_ microspheres was about 60 nm, the thickness of shell was about 9 nm, and the dosage was 0.3 wt%. Gas permeability and porosity are strongly related to each other [15,16]. Since the hollow nanoparticles agglomerate, higher compatibility with film-forming emulsion is necessary. A second approach is to directly prepare film-forming hollow polymer materials. Hollow polymer microspheres have been favored by researchers since their development [17]. The hollow microspheres are a core-shell-structure polymer with a special cavity inside. Compared with solid polymer microspheres, hollow polymer microspheres are lighter, have a lower gas barrier and have been widely used in coatings, inks, leather, paper and potential application prospects [18,19,20]. To better meet the requirements of biodegradability and biocompatibility, natural polymer-based hollow microspheres have attracted attention. Up until now, many types of natural polymers, including chitosan [21,22], starch [23], gelatin [24], pectin [25], polylactic acid [26], casein [27,28,29] and carboxymethyl cellulose [30], have been used to prepare the hollow microspheres. 

Among them, rod-like cellulose nanocrystals have been reported to have excellent mechanical properties, oxidation resistance, dispersion stability in water, and biocompatibility. They have gradually developed into a separate research field of polymer materials [31,32]. CNCs are mainly used as a reinforced filler [33,34] or gas barrier material [35,36] in manufacturing paper, packaging and others. Several hydroxyl groups of the surface of CNCs can be easily modified [37], thereby obtaining a series of modified CNCs and CNCs composites [38,39]. Several reports on CNCs hollow microspheres have been published. For example, Zhang et al. [40] used CNCs and cinnamate-modified CNCs to stabilize O/W Pickering emulsion, and W/O inverse Pickering emulsion to fabricate hydrophobic and hydrophilic polymeric hollow microcapsules (PHMs), respectively. The Pickering emulsion polymerization in the presence of crosslinker inside the emulsion droplets will partition the crosslinked polymers to the oil–water interface, favoring the formation of PHMs. However, to our best knowledge, the current literatures are mainly focused on the regulation of the hollow microsphere structures by adjusting synthesis parameters; very few efforts have been made to directly fabricate CNCs hollow microsphere for coatings, let alone an in-depth investigation of their application on PET fabrics to enhance breathability and moisture permeability. 

This manuscript reports the preparation of cellulose nanocrystals hollow microspheres (HMs) via Pickering emulsion polymerization coupled solvent evaporation. The as-prepared spheres are expected to exhibit excellent film-formable ability, breathability and moisture permeability. The formation mechanism of HMs has been discussed in-depth. Further, this study offers a strategy for fabricating cellulose-based hollow spheres, solving the poor breathability and moisture permeability of polyester fabrics, further expanding the application field of nanocellulose, and improving the value of PET fabrics.

## 2. Materials and Methods

### 2.1. Materials

Cellulose nanocrystals (white powder, the crystallinity degree >75%, L/D: 10~40) without acidic groups were purchased from the University of Maine (Oronoo, ME, USA). γ-methacryloxypropyl trimethoxysilane (A174) was supplied by Shanghai Macklin Biochemical Co., Ltd. (Shanghai, China). Dichloromethane (CH_2_Cl_2_) was obtained from Shanghai Guoyao Chemical reagents Co., Ltd. (Shanghai, China). Ammonium persulfate (APS), n-butyl acrylate (BA), toluene, and absolute ethyl alcohol were supplied by Tianjin Kemio Chemical Reagents Co., Ltd. (Tianjin, China). Vegetable oil exacted from peanut was purchased at the supermarket. All the chemicals were analytical grade and used without further treatment. Deionized (DI) water from a water purification system was used to prepare all aqueous solutions.

### 2.2. Preparation of Modified Cellulose Nanocrystals Particles

Firstly, 0.5 g of CNCs was added to the mixed solution (ethanol: water = 9:1) with ultrasonic dispersion in an ultrasonic cell disintegrator for 15 min at 30 W to mix evenly. The CNCs solution was then added to a 100 mL three-necked round-bottom flask fitted with a digital electric stirrer, a reflux condenser, a thermometer and a constant pressure dropping funnel. This solution was stirred at 300 r/min. When the temperature was raised to 75 °C, 2.5 g of A174 was dropwise added to the flask with an appropriate dropping rate. The reaction was allowed to go on for 2.0 h. Finally, the obtained modified CNCs were precipitated with acetone, washed three times by centrifugation and vacuum dried.

### 2.3. Synthesis of Cellulose Nanocrystals Hollow Microspheres

HMs were prepared in two steps by Pickering emulsion polymerization and solvent evaporation. Firstly, 0.12 g modified CNCs, dichloromethane (20 g) and deionized water (20 g) were homogenized by ultrasonic for 15 min at 10,000 r/min speed to form Pickering emulsion for further use. In a typical procedure, the above Pickering emulsion was added to a 250 mL three-neck flask containing 20 g deionized water, equipped with a mechanical stirrer, a temperature controller, and a condenser. The mixture was stirred at a rate of 300 rpm and then 10 g of APS solution was simultaneously fed to the system. This reaction was carried out for 3.0 h at 75 °C. Secondly, removing the condenser, the solvent was volatilized by stirring at 45 °C for 5 h. The HMs were obtained and then were extracted from the upper emulsion by centrifugation at 8000 r/min. Cellulose nanocrystals core-shell microspheres (CSMs) were also prepared as a control sample in order to investigate the effect of hollow structure on the performance of HMs. They were prepared using the same protocol as CNCs, with the only exception being that 0.1 g BA was added at the first step for preparing CSMs. 

### 2.4. Fabrication Polyester Fabric with Cellulose Nanocrystals Hollow Microsphere Coatings

Before finishing onto the PET fabric’s surface, 20 g/L sodium hydroxide solution was made according to the liquor ratio of 50:1. When the temperature was raised to 80 ℃, the PET fabrics and alkali were mixed by an electromagnetic stirrer for 30 min. The 20 cm × 20 cm fabric pieces were etched with alkali, washed with DI water, and dried in an oven. The obtained PET fabrics containing hydroxyl groups were padded with two dips and nips (95% wet pick-up) in CNCs-based emulsions samples of 70 g/L. After the treatment, the resultant PET fabrics were dried at 80 °C for 3 min and cured at 110 °C for 2 min.

### 2.5. Characterization

Before tests, the samples had to be purified by five cycles of centrifugation–dispersion using absolute ethyl alcohol. After drying, the samples were ground into powder and dried in an infrared-ray oven until they reached a constant weight. Next, KBr was mixed with the same dose of as-produced powders and pressed in a disk-shaped probing sample for FTIR measurements. The chemical structure of the sample powders was then determined by a Spotlight 400 FT-IR spectrometer (PerkinElmer, Waltham, MA, USA) in the spectra range from 4000 cm^−1^ to 400 cm^−1^ with × resolution of 4 cm^−1^, and forward and reverse moving mirrors speed of 10 and 6.2 kHz, respectively. Thermogravimetric analysis (TGA) and differential thermal gravity (DTG) measurement ware carried out by a thermogravimetric analyzer (METTLER TOLEDO TGA/SDTA 851e, Mettler Toledo Technology (China) Co., Ltd., Shanghai, China) with a heating rate of 10 °C/min from 30 to 800 °C in a nitrogen atmosphere. The morphology of the CNCs-based microspheres was characterized using H-600 TEM (Hitachi, Japan) instrument. Before TEM observation, the samples were stained and dried onto carbon-coated copper grids.

The emulsion type was determined by dilution using oil and water once Pickering emulsions were formed. If the emulsion was readily dispersed in the water phase, it was of O/W type, while if the emulsion was dispersed readily in the oil phase, it was classified as a W/O type. Meanwhile, the height of the serum phase (Hs) and the total height of formulation (Ht) were recorded along with the incubation time at room temperature, and the creaming/sedimentation index was reported as (Hs/Ht) × 100. Pickering emulsion was observed using optical microscopy (DM2500M, Leica, Wetzlar, Germany). A drop of Pickering emulsion (50% *v*/*v*) sample was dripped onto the glass slice with a covering slide and then placed on a microscope slide and imaged at 50× or 100× magnifications. 

The as-prepared CNCs-based microspheres emulsions were applied as coating material on PET fabrics, and the morphology and application properties were measured. Atomic force microscopy (AFM) (SPI3800, Seiko Instruments Inc, Japan) was used to study the surface morphology of PET fabrics. The 5 mm × 5 mm fabric pieces samples were placed on the surface of a clean slide. The AFM observation was performed with a 2 × 2 μm scanner in tapping mode. The morphology of PET fabrics was examined using KYKY-2800B SEM instrument (KYKY, Beijing, China) equipped with an energy-dispersive X-ray (EDX) 32 system simultaneously. The samples were sputter-coated with gold to enhance their surface conductivity before scanning at an acceleration voltage of 5 kV. According to GB/T5453-1997 and light industrial standard, e.g., GB/T12704.1/2-2008 and GB/T 21655.2-2009, the breathability was measured with digital fabric air permeability tester (YG461E, Wuhan Guoxin Instrument Co., Ltd., Wuhan, China). For each sample (Φ 10 cm), the test was carried out three times, from which the average result was calculated. According to the AATCC test method 195–2011, wetting time, absorption rate, maximum wetted radius, spreading speed, accumulative one-way transport capability, and overall moisture management capability (OMMC) of PET fabrics were measured by using an MMT (FX3150, TEXTEST, Switzerland). A schematic diagram of the MMT apparatus, schematic of one-way transport and grading table of MMT indices were shown in Figures S1 and S2 and Table S1, respectively. During the test, the same quantity of solution (0.15 g) was applied onto each specimen’s top surface automatically by the instrument. The pump time was 20 s and total test time was 120 s. The test solution then transferred onto the fabrics in three directions: spreading outward on the top surface (inner) of the fabric; transferring through the fabric from the top surface to the bottom surface (outer); spreading outward on the bottom surface of the fabrics. All specimens (8.0 cm × 8.0 cm) were conditioned and tested in standard atmosphere conditions. Based on the signals measured, a set of indices was calculated according to AATCC test method 195–2011 [41,42,43].

## 3. Results and Discussion

### 3.1. Preparation of Cellulose Nanocrystals Hollow Microspheres

**Figure 1:**
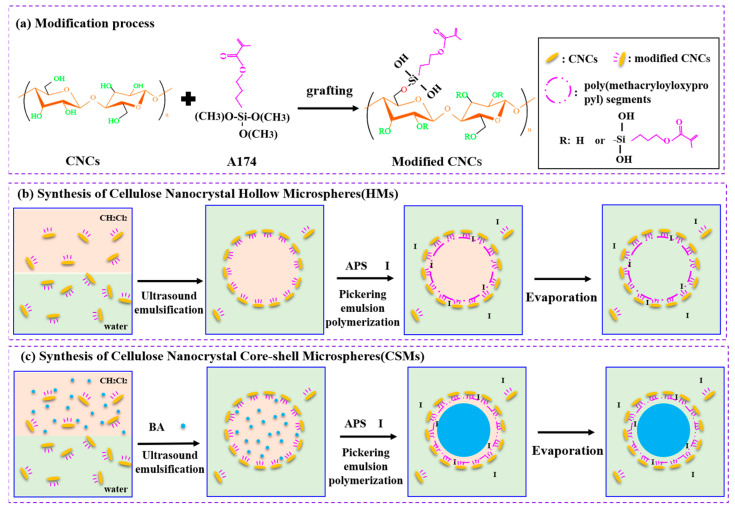
Schematic illustration of (**a**) CNCs modification process, synthesis of (**b**) HMs and (**c**) CSMs through Pickering emulsion polymerization and solvent evaporation process.

Figure 1 outlines the whole process for preparation of HMs. The synthesis strategy involves first forming a stabilizer of modified CNCs particles with proper hydrophobicity and is the key step to control stability of Pickering emulsion and the resultant CNCs-based microspheres. The second step involves Pickering emulsion polymerization followed by solvent evaporation to obtain HMs or CSMs with the cross-linking modified CNCs at the shell.

CNCs can be used as stabilizer for Pickering emulsion to further design functional nanomaterials [44,45,46]. However, the hydrophilic nature of the CNCs restricts their utilization in stabilizing emulsions and dispersion well in a polar organic media [47]. A174 was selected to modify CNCs so that modified CNCs can act as stabilizers in polar organic solvents, as shown in Figure 1a. A174 contains a methacryloyloxypropyl group at one end, which can improve the hydrophobicity of CNCs. The alkoxy groups at the other end of A174 molecules were hydrolyzed to form a silyl hydroxyl group that underwent hydrolysis polycondensation reaction with the hydroxyl group of CNCs. Thus, the modified CNCs particles were achieved with the diminished polarity driving force for stabilizer with relative higher emulsifying ability. The amphiphilic of modified CNCs could be adjusted by controlling the A174 dosage, making a more stable Pickering emulsion at the oil–water interface [48].

Based on the modification process discussed above (Figure 1a), HMs and CSMs were synthesised via Pickering emulsion polymerization with modified CNCs as emulsifier, as depicted in Figure 1b,c. Taking advantage of the polymerization of methacryloyloxypropyl groups on modified CNCs, under the action of initiator, the adjacent modified CNCs on the surface of the Pickering emulsion droplets were polymerized. Thus, the modified CNCs were connected together to form a stable shell layer. Finally, the hollow structure expanded through solvent evaporation. As shown in Figure 1b, the HMs were formed by integrating poly(methacryloyloxypropyl) segments with modified CNCs as shell layer.

In order to investigate the effects of hollow structure on the performance of finished PET fabrics, solid CSMs were also prepared as the control sample. As displayed in Figure 1c, modified CNCs stabilized Pickering emulsion droplets provided a polymerizing place for hydrophobic monomers. In the case, methacryloyloxypropyl group could be co-polymerized with butyl acrylate in present of APS. The resultant CSMs possessed a solid core layer after the subsequent solvent evaporation.

### 3.2. Characterization of CNCs-Based Microspheres

**Figure 2:**
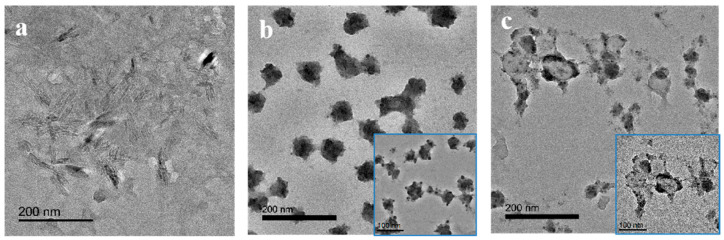
TEM images of (**a**) modified CNCs, (**b**) CSMs, (**c**) HMs. The inset shows the enlarged TEM images of corresponding samples.

The morphology of modified CNCs and CNCs-based microspheres were investigated by TEM. As shown in Figure 2a, the TEM image of modified CNCs revealed rod-like nanoparticles of 60–100 nm length and ~10 nm width. It is clear that CSMs displayed a solid, monodisperse, spherical shape with a diameter of ~60 nm (Figure 2b). Compared with CSMs, the resultant HMs showed an incomplete hollow structure in shape and the average size increased slightly to 60 nm~100 nm, as shown in Figure 2c. It might be related to the poor strength of the shell. During the solvent evaporation process, poly (methacryloyloxypropyl) segments and modified CNCs were entwined together, causing an increase in size and a prominent boundary between the shell and hollow core layer, as shown in Figure 2c inset.

Interestingly, the resultant CSMs and HMs exhibited an apparent nano size that was smaller than the size of modified CNCs. It has been reported that one-dimensional rod-like nanoparticles showed better emulsification efficiency than the spherical zero-dimensional nanoparticles due to the interconnected network formed at the Pickering emulsion interface [40]. Such being the case, this contrast in dimensions can probably be attributed to multilayer modified CNCs cross-linked with the shell. Additionally, there are cross-linked black silica particles on CNCs-based microspheres due to the condensation of silanol groups on the surface of modified CNCs resulting in some agglomeration of resultant HMs and CSMs.

**Figure 3:**
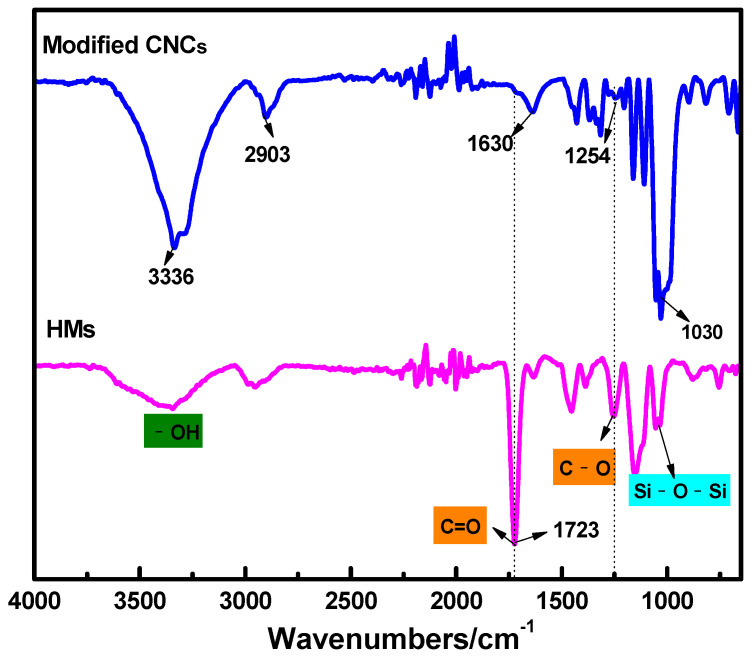
FT-IR spectra of modified CNCs (blue line) and HMs (pink line) for appropriate.

The successful preparation of modified CNCs and HMs was confirmed by FTIR spectra, as shown in Figure 3. The characteristic bands around 3336 cm^−1^, 1430 cm^−1^ and 1319 cm^−1^ were attributed to the -OH stretching, C-H bending vibration, in-plane bending stretching of -CH_2_ of CNCs, consistent with the literature [48]. The absorption peaks at 896 cm^−1^and 1630 cm^−1^ were attributed to Si-OH bending and the C=C stretching of A174, respectively, proving that CNCs had been successfully modified by A174. The absorption peak at 1030 cm^−1^ corresponded to symmetrical stretching vibration of Si-O-Si, further confirming the formation of SiO_2_. It is well consonant with the TEM results. In comparison to the FTIR spectrum of modified CNCs, the spectrum of HMs showed obviously additional vibrations at 1723 cm^−1^ and 1254 cm^−1^, which could be assigned to stretching vibration of C=O and C-O of A174, respectively. The stretching vibration at 1630 cm^−1^ weakened and that at 1723 cm^−1^ sharpened because of the significantly increased ester groups on the surface of modified CNCs. In addition, the absorption peak around 2903 cm^−1^ belongs to the methylene(-CH_2_-) and methyl (-CH_3_) groups of poly (methacryloyloxypropyl) segments were wider, indicating that the methacryloyloxypropyl groups on the adjacent modified CNCs particles were polymerized for the shell layer of HMs. 

**Figure 4:**
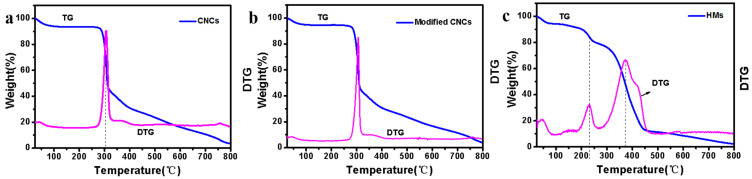
Thermogravimetric analysis (TGA, in blue) and differential thermogravimetry (DTG, in pink) curves of (**a**) CNCs, (**b**) modified CNCs and (**c**) HMs.

In order to further verify the formation of hollow structure and increased thermal behavior of HMs, the thermal decomposition behavior of CNCs, modified CNCs and HMs was investigated by TGA-DTG technique at a heating rate 10 °C/min under N_2_ flow, as illustrated in Figure 4. It can be seen from the figure that all the samples exhibited a slight mass loss before reaching 200 °C, which was caused by the vaporization of free water and bound water. As shown in Figure 4a and Figure 4b, the CNCs and modified CNCs powders showed the same thermal degradation curves. They exhibited one sharp degradation stage at 200–320 °C, which was related to the weight-loss of CNCs framework [49]. The maximum thermal decomposition rate of the CNCs and modified CNCs were 308 °C and 313 °C, respectively (Figure 4a,b). This was due to the condensation of the silanol groups on the modified CNCs, resulting in higher thermal stability [50]. It also further demonstrated that CNCs were modified by A174. Based on the residual mass, the grafting degree of the A174-modified CNCs could be calculated as 15.4%. The TGA-DTG curves of HMs were shown in Figure 4c. The thermal decomposition process of HMs went through three stages. The first stage (25–150 °C) was the weight-loss of water. The second stage (150–270 °C) was mainly associated with the poly(methacryloyloxypropyl) segment decomposition. The third stage was the thermal degradation of the shell of HMs in the range of 270–460 °C, with the fastest thermal decomposition temperature being around 360 °C. It could be concluded that poly(methacryloyloxypropyl) segments underlining the modified CNCs provide the shell of HMs with strong heat resistance. Additionally, it was also found that the curves of HMs were clearly different from modified CNCs although modified CNCs and HMs had the same composition. It was presumably attributed to the hollow structure and the debasing dynamics of the interface effect between constituent polymers of the shell and inner cavity [18]. Overall, the results revealed that A174 was grafted on CNCs and HMs was further successfully conducted.

### 3.3. Modified Cellulose Nanocrystals as Pickering Emulsion Stabilizer

**Figure 5:**
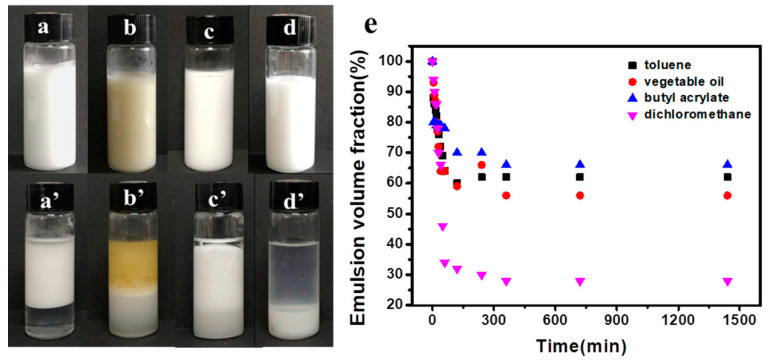
Appearance of Pickering emulsion stabilized by modified CNCs standing for 0 h (**a**–**d**) and 24 h (**a’**–**d’**) in various oil dispersion: (**a**,**a’**) toluene, (**b**,**b’**) vegetable oil, (**c**,**c’**) butyl acrylate, (**d**,**d’**) dichloromethane, and (**e**) their emulsion volume fraction. The concentration of modified CNCs was fixed at 0.3% (*w*/*v*). The oil/water ratio was 1:1.

Hydrophobic modification of CNCs is necessary to obtain O/W Pickering emulsions [51,52,53]. As shown in Figure 5, Pickering emulsions stabilized by modified CNCs in different oil dispersions were examined to find out if the modified CNCs exhibited their emulsifying function. The properties of oil phases directly influenced the formation of Pickering emulsion [54]. Four typical oils with different polarity were selected for investigation, including butyl acrylate, peanut oil, dichloromethane and toluene [55]. The dielectric constant, viscosity and density parameters of different oil phases were shown in Table S2. Peanut oil and toluene are weak polar oil phases, while dichloromethane is a strong polar oil phase. Butyl acrylate is a polar monomer without dielectric constant. After homogenization (Figure 5a–d) and further standing for 24 h (Figure 5a’–d’), it was seen from the appearance of the emulsion that the one using dichloromethane as the oil phase was located in the lower part of the container as the density of dichloromethane is greater than that of water, resulting in the sinking of the formed Pickering emulsion under the water phase. On the contrary, the formed O/W emulsion using the other oil dispersions floats on the aqueous phase. As depicted in Figure 5e, the stability of Pickering emulsion stabilized by modified CNCs for weak polar oil were much better than that for strong polar solvents. When the oil phase was BA, the emulsion volume fraction was highest and stability of obtained Pickering emulsion was best. This increased stability could be attributed to stronger interactions between the residual conjugate domains in BA and methacryloyloxypropyl of modified CNCs [56]. In addition, the silica particles on modified CNCs shown in TEM could play a role as stabilizer [57], improving the stability. The result of emulsion volume fraction was consistent with standing stability.

**Figure 6:**
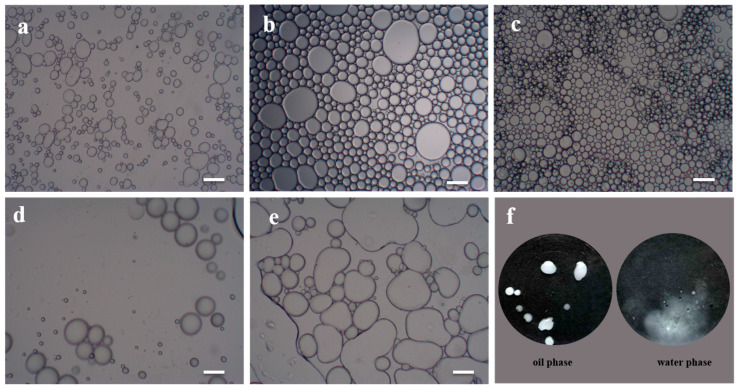
Optical microscopy images of Pickering emulsion stabilized by modified CNCs prepared in different A174 concentrations: (**a**) 1.2%, (**b**) 1.8%, (**c**) 2.4%, (**d**) 3.0%, (**e**) 3.6%. (**f**) the type of Pickering emulsion. The scale bar was 10 μm.

To the best of our knowledge, colloidal stability is of paramount importance for the stabilizer in order to avoid aggregation [58,59]. Hence, the amphiphilic of modified CNCs was controlled by changing the A174 usages. Optical microscopy was further employed to observe the microstructure of Pickering emulsion stabilized by modified CNCs at different A174 concentrations, as displayed in Figure 6. The size distribution of Pickering emulsion droplets in different A174 concentrations were counted by Image-Pro Plus software, as presented in Figure S3. All the emulsions were O/W types (Figure 6f). When the A174 concentration was lower than 2.4%, the stabilized Pickering emulsion droplets were more spherical and uniform in size of 9.7 μm, 32.73 μm, respectively. With the increase of A174 concentration, the emulsion droplets were small and uniform. According to Stokes’ law, the smaller the emulsion droplet, the more stable the emulsion [60]. Since the hydrophobic modification of CNCs is insufficient at low modifier concentrations and cannot stabilize the oil and water phases, the emulsion droplets were flat and large in size and had poor stability [61]. At higher modifier concentrations(>2.4%), the hydrophilic modified CNCs were less covered on the oil–water interface, which dramatically enhanced the interface tension and decreased the stability of the emulsion [62], making the size distribution broader and wider (Figure S3d,e). Thus, modified CNCs at 2.4% A174 demonstrated excellent emulsifying properties, forming a stable Pickering emulsion with mean diameter of 29.1 μm. In order to obtain HMs through Pickering emulsion polymerization and solvent evaporation, the oil phase was required to be easily volatile. Hence, dichloromethane was selected for the experiment.

### 3.4. Cellulose Nanocrystals Hollow Microspheres Finished PET Fabrics 

**Figure 7:**
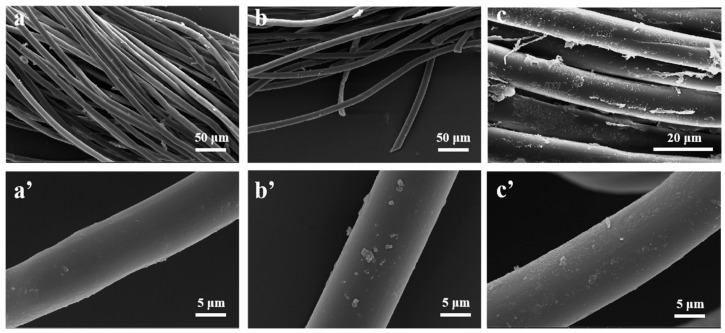
SEM images of (**a**,**a’**) the pristine PET, (**b**,**b’**) CSMs, and (**c**,**c’**) HMs finished PET fabrics.

Nanocellulose is often used as anti-wrinkle finishing for cotton fabrics [62], sustainable wound dressing for nonwoven cotton [63], hydrophilic modification of polyester fabric [6], but it is rarely reported that nanocellulose acts as a moisture permeable and breathable finishing agent. The CNCs-based emulsions were finished onto PET fabrics. SEM and AFM were employed to characterize the surface morphology and surface roughness of pristine PET-, CSMs- and HMs-finished PET fabrics, as shown in Figure 7 and Figure S4. It was found that the pristine PET fabrics had a smooth surface with RMS of 1.984 nm (Figure S4a,a’) and average diameter of about 11.5 µm (Figure 7a). After post-treated with CSMs and HMs, the diameter of the fibers increased to 15 µm (Figure 7b) and 13 µm (Figure 7c), respectively. As shown in Figure 7b, it is noted that CSMs coated PET fabric had RMS of 4.965 nm (Figure S3b,b’) and maintained a smooth surface, which further supported the fact that CNCs-based emulsion had good film-forming ability and solid core-shell microspheres enabled them to form flat and dense coatings. Compared with CSMs-finished PET fabrics, there was an apparent morphology change in HMs-finished PET fabrics, as shown in Figure 7c and Figure S4c,c’. Homogeneous protrusions were randomly distributed on the surfaces of HMs-finished PET fabrics. In order to confirm from the protrusions if it was the HMs coatings, an elemental analysis of the samples was performed by EDX; the results were shown in Figure S5. As a typical spectrum shows in Figure S4, carbon, oxygen, and silicon existed. The amount of silicon was associated with A174 modification CNCs. The corresponding RMS of HMs finished PET fabrics was 7.033 nm. All the results proved that homogeneous protrusions on PET fabric surface were HMs coatings, which were expected to contribute to the correlation between the hollow structure and PET fabric’s breathability and moisture permeability.

### 3.5. Breathability and Moisture Permeability of PET Fabrics

**Figure 8:**
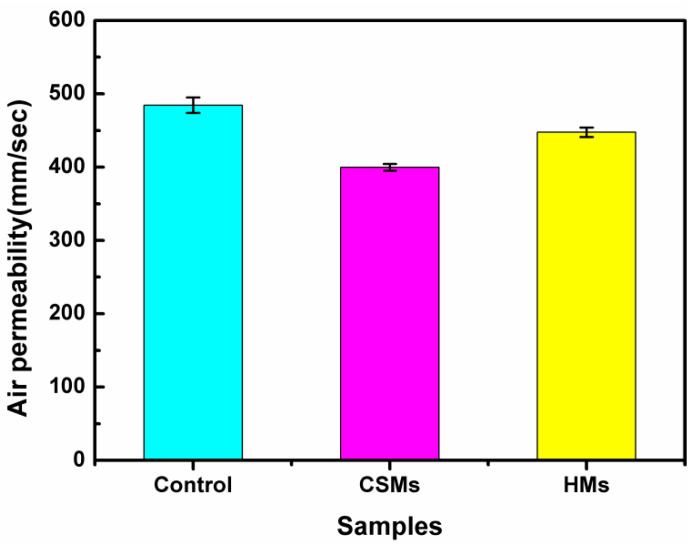
Air permeability results of the pristine PET, and CNCs-based emulsions finished PET fabrics.

The as-prepared emulsions formed coatings on the PET fabric surface in the finishing process to further identify the use of CNCs-based emulsions. Figure 8 shows the air permeability results of pristine PET fabrics, as well as the PET fabrics finished by CSMs and HMs emulsions. The pristine PET fabrics had an air permeability value of 485 mm/sec, as compared with CSMs- and HMs-treated PET fabrics, with an air permeability value of 400 mm/s and 447 mm/s, respectively. The decrease in air permeability was attributed to the presence of thin coatings on the PET fabric surface, which caused an increase in the permeation route and barrier for the gas molecules [64]. Compared with the PET fabrics finished by CSMs, it can be clearly seen that the air permeability of PET fabrics finished by HMs was higher and only slightly lower than pristine PET fabrics. The excellent air permeability was contribution to their hollow structure, which could reduce the flow resistance of gas molecules. Conversely, the decrease in the PET fabrics treated with CSMs was due to the densification of the coatings on the surface of PET fabrics, just as that SEM presented (Figure 7b). Additionally, the moisture management tester (MMT) was used to characterize the liquid flow state and the water transport characteristics of the PET fabrics [9]. Table 1 shows the detailed data.

The presence of only a few hydrophilic groups on the molecular chains enhanced the wetting time of the top and bottom surfaces of pristine PET fabrics with a max wetting radius of 5 mm. The wetting time, absorption rate, max wetted radius and spreading speed of CSMs and HMs finished PET fabrics were significantly better than that of pristine PET fabrics. These were attributed to the hydrogen bonding between water molecules and hydroxyl groups of modified CNCs. Compared with CSMs-finished PET fabrics, HMs-finished PET fabrics represented shorter wetting time and higher absorption rates on the bottom surface. Absorption rates increased from <0.9 mm/s to >1.3 mm/s on the top and bottom surfaces, while the max wetted radius was extended from 10 mm to 10–25 mm. According to AATCC test method 195–2011, the test indices were graded and converted from value to grade based on a five-grade scale (1–5), as shown in Table S1 [65]. Fingerprints of moisture management properties of CSMs- and HMs-finished PET fabrics are shown in Figure S6. Figure S6a shows that the top wetting time and absorption rate of CSMs-finished PET fabrics was in good grade to a very good grade, while Figure S6b for HMs-finished PET fabrics sample fell in the good to fair grade. The top spreading speed (mm) of the CSMs finished PET fabrics sample fell in poor grade, while HMs finished PET fabrics sample was in fair grade. The bottom wetting time and absorption rate of CSMs finished PET fabrics fell into the fair to poor grade while HMs-finished PET fabrics sample was in the good to very good grade. This result indicated that when the developed HMs-finished PET fabrics absorbed moisture or sweat from the top surface, it could be transferred to the outer surface at a relatively fast speed.

One-way transport capacity forms a very important direct indicator of the moisture absorption and perspiration capacity of the fabrics. The absorbed moisture or sweat on the fabrics went from the inner surface, that is in touch with the human skin, to the outside (the surrounding environment) [2,5]. If one-way transport capacity was high, it means the moisture evaporates at very fast speed. However, it would cause the moisture to remain on the inner surface of the fabrics for a longer time, causing a clammy feeling and discomfort [8,66]. The one-way transported index data (Table 1) reflected that both the untreated PET fabric and CSMs-finished PET fabrics showed a negative one-way transported index, indicating poor moisture transport performance in the vertical direction. The HMs-finished PET fabrics showed an excellent unidirectional transport index (936.33%). On the one hand, it was attributed to the fact that the hydroxyl groups on the modified CNCs of the HMs shell have strong water absorption. On the other hand, the discontinuous cavity of HMs coatings reduced the barrier of moisture transport and enhanced the capillary action on the fiber surface. The HMs-finished PET fabrics exhibited excellent grade of one-way water transmission (Figure S6), which further proved that sweat could wet the inner surface of the fabric and transport it in the outward direction quickly. Combining all the indices, the overall moisture management capability (OMMC) of the HMs-finished PET fabrics was found to be 0.723, reaching a very good grade. Thus, HMs-finished PET fabrics are moisture management fabrics, while CSMs-finished PET fabrics are fast absorbing and slow drying. This provides a moisture-wicking finishing agent for PET fabrics.

**Figure 9:**
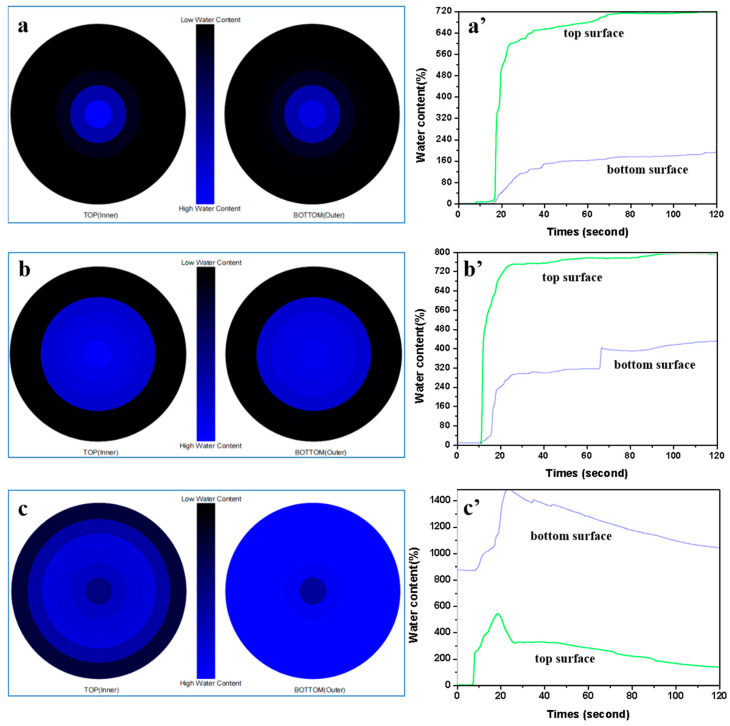
Water location (**a–c**) and water content vs. time (**a’–c’**) of (**a,a’**) the pristine PET, (**b,b’**) CSMs and (**c,c’**) HMs -finished PET fabrics.

Figure 9 represents the moisture content and water location versus time for the top and bottom surfaces of PET fabrics. During the test, the machine dispensed test solution onto the top of the fabric. The test solution then transferred onto the fabrics in three directions: spreading outward on the top surface (inner) of the fabric; transferring through the fabric from the top surface to the bottom surface (outer); spreading outward on the bottom surface of the fabrics. The blue area and the black area represent the wet and dry parts. The closeness of the blue line to the green line determined the transmission performance. As shown in Figure 9a, the max wetted radius was limited to <5 mm. The water dispersion on the pristine PET fabrics remained on the top surface for 8.16 s, and no liquid moisture was transferred to the bottom surface until 16.41 s (Figure 9a’). The moisture content versus time curve on the top surface of the pristine PET fabrics was very steep with a very high initial slope, indicating a very high absorption rate. However, a relatively lower absorption rate was on the bottom surface because of the poor transmission performance from top surface to bottom surface. Therefore, the water content on the bottom surface was very low, as were the absorption rate, and wetting radius. The CSMs-finished PET fabrics showed similar results to the pristine PET fabrics. The moisture remained on the top surface for 10 s before it began to transfer to the bottom surface (Figure 9b), and it was fully wetted to a radius of 10 mm (Figure 9b’). The bottom surface exhibited a steep initial slope before 20 s, indicating that it had a slightly higher absorption at the presence of CSMs coatings. It could be found that the moisture permeability of CSMs-finished PET fabric was greater than that of the pristine PET fabric due to a higher number of hydrophilic hydroxyl groups on the surface of CSMs, which was helpful for PET fabrics to adsorb and transfer water vapor molecules. However, the HMs-finished PET fabrics had a short water retention time on their surface and were completely wetted with a radius of 25 mm (Figure 9c).

As shown in Figure 9c’, the blue line is above the green line, which means the water transmission from the inner part to the outer part increased and the water content on the bottom surface was significantly higher than that on the top surface at 20 s. There was a sawtooth curve and larger slope of the lines after 20 s, which indicated higher evaporation performance. Due to the hydrophilic and hollow structure characteristics, HMs coatings quickly absorbed the water molecules and transferred water quickly. It was implied that the fabric side coated with HMs give the wearer a drier feeling on the skin even after perspiration because of the sweat transportation to the other side, consistent with the analysis shown in Table 1.

## 4. Conclusions

Cellulose nanocrystal hollow microspheres coatings were successfully synthesized via Pickering emulsion polymerization and solvent evaporation. First, well-emulsified modified CNCs were synthesized using 2.4% of A174 by controlling the interface activity of the CNCs surface. During Pickering polymerization, modified CNCs were anchored on the surface of latex particles to form 60 nm~100 nm of HMs. The interaction and stabilizing mechanism of modified CNCs were highlighted for Pickering emulsion, which guided the controlled structure and morphology of CNCs-based composite materials. More importantly, the as-obtained HMs had film-formable property and the coatings exhibited breathability and moisture permeability. The HMs-finished PET fabrics exhibited 447 (mm/s) air breathability and relatively higher water transport performance (one-way transport index = 936.33%, OMMC = 0.72). Thus, these results provided a new method to manufacture breathability and moisture wicking functionality products.

## Figures and Tables

**Table 1:** Moisture management of PET fabrics.

Index		Pristine PET	CSMs Finished PET	HMs Finished PET
Wetting time (s)	Top	8.16	9.75	6.38
	Bottom	16.41	10.88	7.59
Absorption rate (%/s)	Top	35.21	47.79	43.66
	Bottom	8.50	18.56	38.92
Max wetted radius manufacture breathability and manufacture breathability and (mm)	Top	5.0	10.0	10.0
	Bottom	5.0	10.0	25.0
Spreading speed (mm/s)	Top	0.59	0.90	1.34
	Bottom	0.30	0.85	2.71
One-way transported index (%)	-	−445.60	−378.11	936.33
The overall moisture management capability	-	0	0.024	0.723

## Data Availability

The data presented in this study are available on request from the corresponding author.

## Supplementary Materials

The following supporting information can be downloaded at: https://www.mdpi.com/article/10.3390/polym14245345/s1, Figure S1: Schematic diagram of moisture management tester apparatus: (a) sweat gland; (b) top sensor; (c) fabric inner (next to skin) side; (d) fabric outer side; (e) bottom sensor; (f) copper ring; Figure S2: Schematic of one-way transport capability, top surface absorption rate and spreading speed parameters; Table S1: Grading table of MMT indices according to AATCC test method 195–2011; Figure S3: The size distribution of the Pickering emulsion droplets stabilized by modified CNCs prepared in different A174 concentrations: (a) 1.2%, (b) 1.8%, (c) 2.4%, (d) 3.0%, (e) 3.6%, counting by Image-Pro Plus software. Figure S4: AFM planar graphs of (a, a’) the pristine PET, RMS = 1.984 nm, (b, b’) CSMs, RMS = 4.965 nm and (c, c’) HMs-finished PET fabrics, RMS = 7.033 nm; Figure S5: X spectra of HMs coatings on the top surface of PET fabrics; Figure S6: Fingerprints of Moisture management properties of (a) CSMs-finished PET fabrics, (b) HMs-finished PET fabrics.
