# Supplementary material for: Breathability and Moisture Permeability of Cellulose Nanocrystals Hollow Microsphere Coatings for PET Fabrics
Source: Polymers (Basel). 2022 Dec 7;14(24):5345. doi: 10.3390/polym14245345 (PMC9788502; doi:10.3390/polym14245345)
Supplement: Supplementary file 1 [file polymers-14-05345-s001.zip › polymers-1930316-supplementary.pdf]

Supplementary Material

Breathability and Moisture Permeability of Cellulose Nanocrystals Hollow Microsphere Coatings for PET Fabrics

Fan Zhang [1,2,3,*], Bingyao Song [1,3], Yilin Li [1,3], Yingying Zhou [1,3,4], Yanbing Wang [1,3], Qunna Xu [4] and Jianzhong Ma [2,4]

[1] School of Textile Science and Engineering, Xi'an Polytechnic University, Xi'an 710048, China
[2] Shaanxi Collaborative Innovation Centre of Industrial Auxiliary Chemistry & Technology, Shaanxi University of Science & Technology, Xi'an 710021, China
[3] Key Laboratory of Functional Textile Material and Product, Xi'an Polytechnic University, Ministry of Education, Xi'an 710048, China
[4] College of Bioresources Chemical and Materials Engineering, Shaanxi University of Science and Technology, Xi'an 710021, China
* Correspondence: zhangfan@xpu.edu.cn; Tel.: +86-029-82330365

Citation: Zhang, F.; Song, B.; Li, Y.; Zhou, Y.; Wang, Y.; Xu, Q.; Ma, J. Breathability and Moisture Permeability of Cellulose Nanocrystals Hollow Microsphere Coatings for PET Fabrics. *Polymers* **2022**, *14*, x. https://doi.org/10.3390/xxxxx

Academic Editors: Xuefeng Zhang, Weiqi Leng

Received: 6 September 2022
Accepted: 5 December 2022
Published: 7 December 2022

Publisher's Note: MDPI stays neutral with regard to jurisdictional claims in published maps and institutional affiliations.

Copyright: © 2022 by the authors. Submitted for possible open access publication under the terms and conditions of the Creative Commons Attribution (CC BY) license (https://creativecommons.org/licenses/by/4.0/).

2.5. Characterization

According to AATCC test method 195-2011, wetting time, absorption rate, maximum wetted radius, spreading speed, accumulative one-way transport capability, and overall moisture management capability (OMMC) of PET fabrics were measured by using a MMT (FX3150, TEXTEST, Switzerland). A schematic diagram of the MMT apparatus is given in Figure S1 [41]. The principle is based on the fact that when there is moisture transport in the fabric, the contact electrical resistance of the fabric will change and the value of the resistance change depends on two factors: the components of the liquid and the water content in the fabric. The liquid components are fixed, so that the electrical resistance measured is related to the water content in the fabric [42]. A total of 9 g of sodium chloride was dissolved in 1 L of distilled water to achieve 16 ± 0.2 mS of solution conductivity and the solution was dropped onto the fabric's top surface. The test solution will then transfer onto the fabrics in three directions: spreading outward on the top surface (inner) of the fabric; transferring through the fabric from the top surface to the bottom surface (outer); spreading outward on the bottom surface of the fabrics. The schematic of one-way transport capability, top surface absorption rate and bottom spreading speed parameters as show Figure S2 [43]. During the test, the same quantity of solution (0.15 g) was applied onto each specimen's top surface automatically by the instrument. The test liquid is dispensed through the gland to the top surface of the fabric sample, which is designed as an inner surface that will be in touch with the human skin. The pump time is 20 s and total test time was 120 s. All specimens (8.0 cm × 8.0 cm) were conditioned and tested in standard atmosphere conditions. Based on the signals measured, a set of indices is calculated according to AATCC test method 195-2011; the indices are graded and converted from value to grade based on a five grades scale (1–5). The five grades of indices represent the following: 1 – poor; 2 – fair; 3 – good; 4 – very good; 5 – excellent. Table S1 shows the range of values converted into grades.

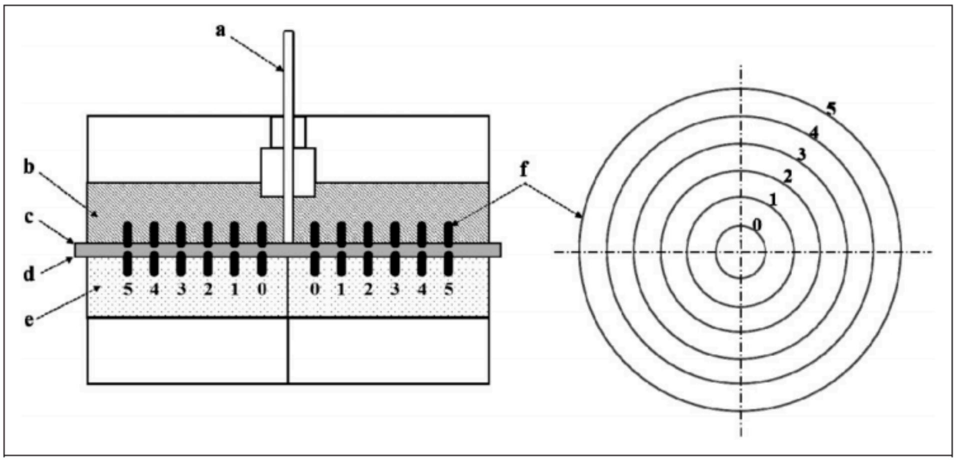

Figure S1. Schematic diagram [1] of moisture management tester apparatus: (a) sweat gland; (b) top sensor; (c) fabric inner (next to skin) side; (d) fabric outer side; (e) bottom sensor; (f) copper ring [41].

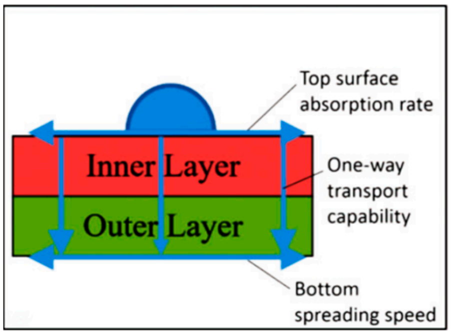

Figure S2. Schematic of one-way transport capability, top surface absorption rate and spreading speed parameters [43].

Table S1. Grading table of MMT indices [65] according to AATCC test method 195-2011.

Index		Grade				
		1	2	3	4	5
Wetting time(s)	Top	≥120	20–119	5–19	3–5	<3
	Bottom	≥120	20–119	5–19	3–5	<3
Absorption rate(%/sec)	Top	0-9	10–29	30–49	50–100	>100
	Bottom	0-9	10–29	30–49	50–100	>100
Max wetted radius(mm)	Top	0-7	8–12	13–17	18–22	>22
	Bottom	0-7	8–12	13–17	18–22	>22
Spreading speed(mm/sec)	Top	0.0-0.9	1.0–1.9	2.0–2.9	3.0–4.0	>4.0
	Bottom	0.0-0.9	1.0–1.9	2.0–2.9	3.0–4.0	>4.0
One-way transported index (%)		<-50	-50–99	100–199	200–400	>400
verall moisture management capability (OMMC)		0.00–0.19	0.20–0.39	0.40–0.59	0.60–0.80	>0.80

Table S2. The dielectric constant, viscosity and density parameters of different oil phases.

Index \ Oil	Toluene	Peanut oil	Dichloromethane	BA
Dielectric constant	2.4	3	9.1	-*
Viscosity (MPa·s)	0.59	10~12	0.43	0.81
Density (g/mL) at 25 °C	0.866	0.8~0.9	1.325	0.8988

* unaccessed data.

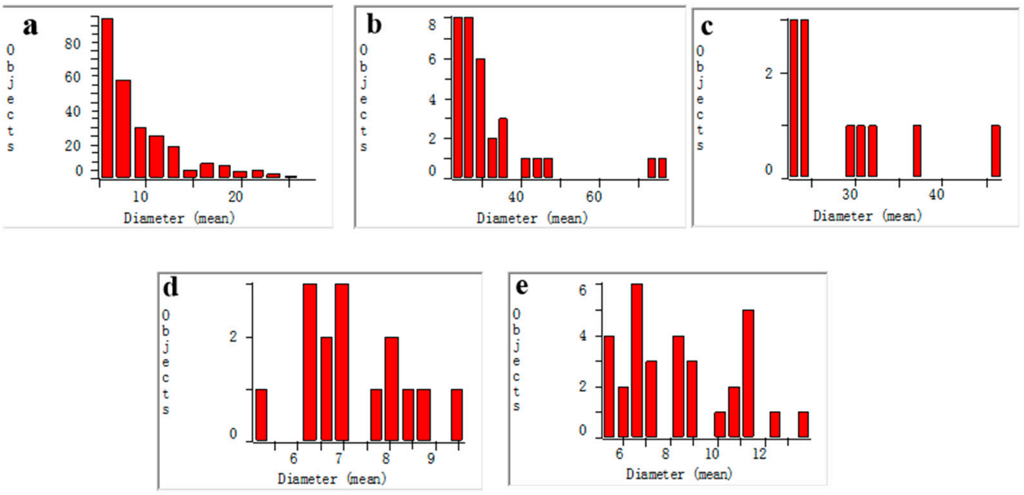

Figure S3. The size distribution of the Pickering emulsion droplets stabilized by modified CNCs prepared in different A174 concentrations:(a) 1.2%, (b) 1.8%, (c) 2.4%, (d) 3.0%, (e) 3.6%, counting by Image pro plus software.

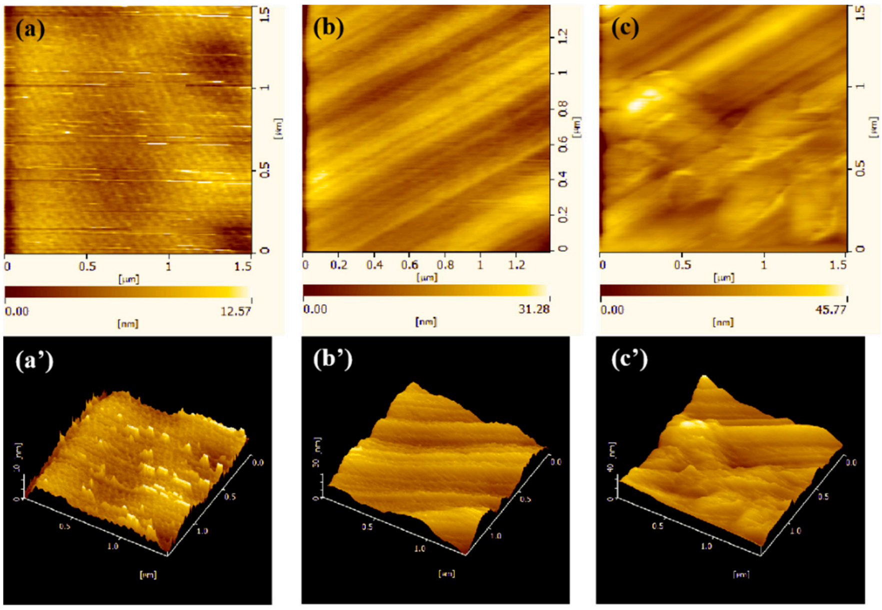

Figure S4. AFM planar graphs of (a, a') the pristine PET, RMS=1.984 nm, (b, b') CSMs, RMS=4.965 nm and (c, c') HMs finished PET fabrics, RMS=7.033 nm;.

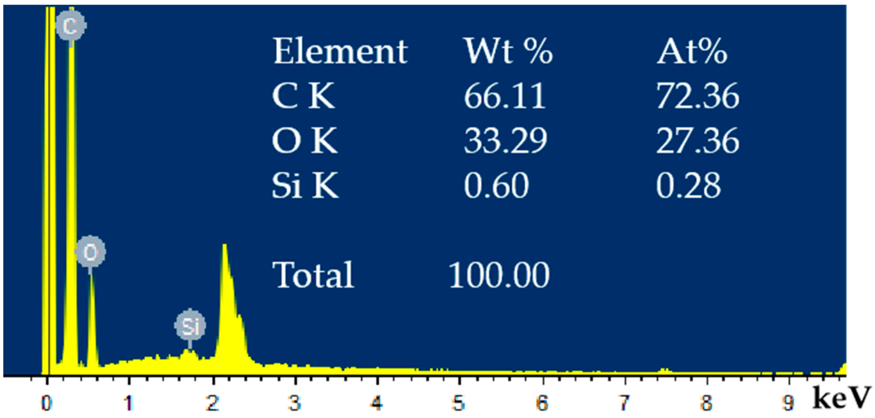

Figure S5. X spectra of HMs coatings on the top surface of PET fabrics.

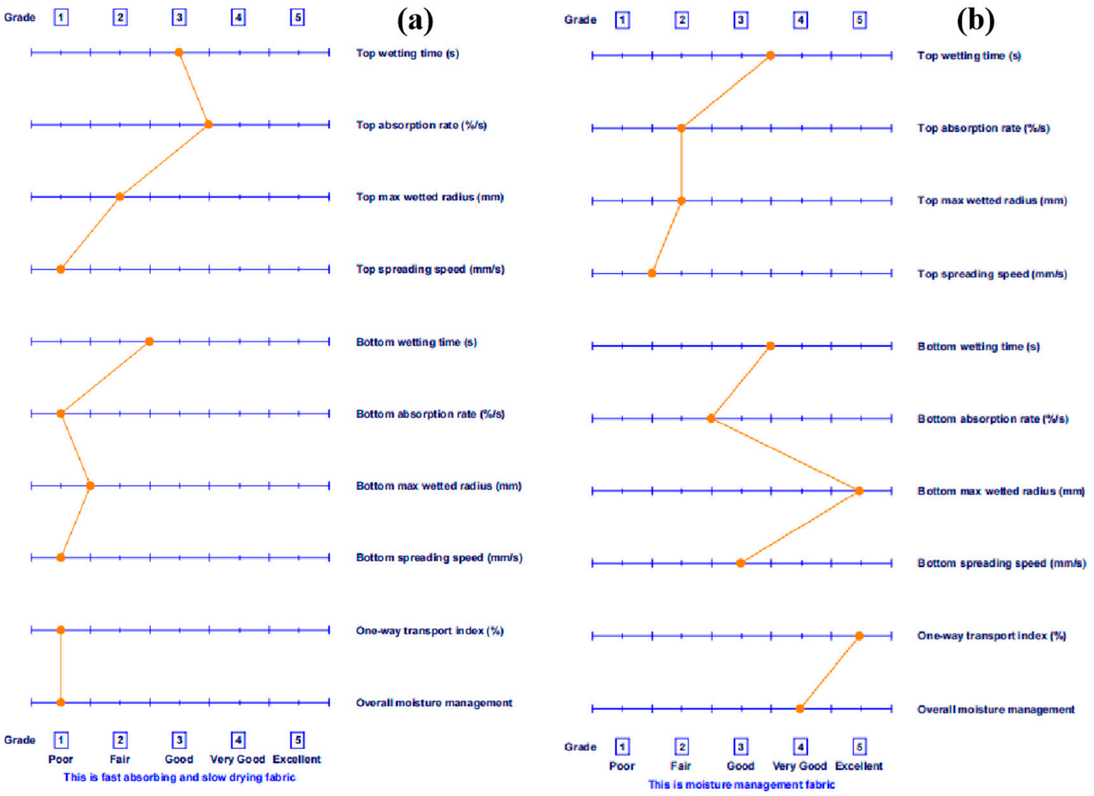

Figure S6. Fingerprints of Moisture management properties of (a) CSMs finished PET fabrics, (b) HMs finished PET fabrics.

Reference

41. Jung, J.L.; Dong, S.J. Evaluation of liquid moisture management properties on hemp woven fabrics treated with liquid ammonia. Text. Res. J. 2017, 87, 1752–1764.
42. Li, Y.; Xu, W.; Yeung, K.W. Moisture Management of Textiles. U.S. Patent 6,499, p.338 B2, 2000.
43. Huang, J.F.; New, J.; Tham, J.B.; Tok, A. Novel moisture management test of polyethylene terephthalate and nylon fabric under stretching and surface patterning. Text. Res. J. 2018, 88, 69–79.
65. McQueen, R.H.; Batcheller, J.C.; Mah, T.; Hooper, P.M. Development of a protocol to assess fabric suitability for testing liquid moisture transport properties. J. Text. Inst. 2013, 104, 900–905.
